# Fetal Hepatosplenomegaly: Stepwise Diagnostic Framework, Diagnostic Approach to Fetal Hepatosplenomegaly

**DOI:** 10.3390/jcm15166274

**Published:** 2026-08-13

**Authors:** Maria Mazek, Michał Ciebiera, Diana Massalska

**Affiliations:** 1 Department of Obstetrics, Perinatology, Gynecology and Reproductive Medicine, Medical University of Warsaw, 02-091 Warsaw, Poland; mariamazek.md@gmail.com; 2 Warsaw Institute of Women’s Health, 00-189 Warsaw, Poland; 3 Department of Gynecology, Obstetrics and Gynecological Endocrinology, Centre of Postgraduate Medical Education, 01-813 Warsaw, Poland

**Keywords:** fetal hepatosplenomegaly, fetal hepatomegaly, fetal splenomegaly, congenital infection, non-immunological fetal hydrops, prenatal diagnosis, prenatal ultrasound

## Abstract

**Background/Objectives:** Fetal hepatosplenomegaly is an uncommon but clinically significant prenatal finding associated with a wide range of heterogeneous conditions, including congenital infections, fetal anemia, genetic syndromes, metabolic disorders, and other, less common abnormalities. Because of its nonspecific presentation and diverse etiologies, it represents a diagnostic challenge that requires a structured, multidisciplinary approach. This review aims to present a practical, clinically oriented, stepwise approach to the prenatal evaluation of fetal hepatosplenomegaly, integrating current evidence to facilitate differential diagnosis and guide prenatal management. **Methods:** A comprehensive literature review was performed, focusing on ultrasound diagnosis, differential diagnosis, genetic evaluation, and prenatal management strategies in fetuses with hepatosplenomegaly. **Results:** The prenatal detection of hepatosplenomegaly should prompt a detailed ultrasound assessment, including the confirmation of organ enlargement, evaluation of associated structural abnormalities, Doppler studies, and screening for signs of fetal anemia, infection, hydrops fetalis, or cardiac dysfunction. The diagnostic workup should begin with targeted maternal and fetal investigations directed toward the most common causes. In unresolved cases, genetic testing—including chromosomal microarray analysis and next-generation sequencing—may help establish the diagnosis. The preservation of biological material obtained during invasive procedures should be considered to allow future molecular analyses if new clinical findings emerge. The management depends on the underlying etiology and may include fetal therapy, maternal treatment, or specific interventions in selected genetic and metabolic conditions. **Conclusions:** Fetal hepatosplenomegaly requires a systematic diagnostic strategy that integrates ultrasound, laboratory, and genetic assessment. A structured approach may improve diagnostic accuracy, optimize prenatal counseling, and facilitate the timely management of potentially treatable conditions.

## 1. Introduction

Fetal hepatosplenomegaly is an uncommon but clinically important prenatal finding that may represent the first manifestation of a broad spectrum of fetal disorders. The differential diagnosis encompasses congenital infections, fetal anemia, genetic disorders, metabolic diseases, hematologic conditions, and other, rarer heterogeneous causes [1,2]. Although some underlying conditions carry a poor prognosis, others are potentially treatable in utero—such as fetal anemia managed with intrauterine transfusions—or benefit from optimized perinatal management, including delivery at a tertiary referral center with access to specialized neonatal care [1,3]. Consequently, early etiological diagnosis has important implications for fetal surveillance, prenatal counseling, pregnancy management, recurrence risk assessment, and postnatal follow-up [1,2].

Despite advances in prenatal ultrasound, molecular genetics, and fetal therapy, the prenatal evaluation of hepatosplenomegaly remains challenging. Routine fetal anatomical assessment does not include the direct measurement of the liver or spleen [4,5,6], and no universally accepted prenatal definition of hepatomegaly or splenomegaly currently exists [1,2]. Published reference ranges vary according to measurement technique and reference population, and mild organ enlargement may occasionally reflect a physiological variation rather than a pathology. Furthermore, fetal hepatosplenomegaly is a nonspecific sonographic sign rather than a diagnosis itself, making its interpretation highly dependent on associated ultrasound findings, gestational age, maternal history, and complementary laboratory investigations.

Recent developments in prenatal diagnostics have greatly expanded the ability to identify the underlying causes of fetal abnormalities [7]. High-resolution ultrasound, Doppler assessment, polymerase chain reaction (PCR)-based diagnosis of congenital infections, chromosomal microarray analysis (CMA), and next-generation sequencing (NGS) have significantly improved diagnostic yield [8]. At the same time, fetal therapeutic options continue to evolve, expanding the range of conditions amenable to prenatal intervention [9]. These advances highlight the need for a rational diagnostic strategy that prioritizes common and potentially treatable conditions before proceeding to more specialized investigations.

Although several reviews summarized individual etiologies of fetal hepatosplenomegaly, most are organized as descriptive lists of diseases rather than practical guides for clinical management [1,2]. To date, no comprehensive, evidence-based framework has integrated current diagnostic methods into a practical, stepwise clinical pathway for the prenatal evaluation of fetal hepatosplenomegaly.

## 2. Aim of the Study

The aim of this study is to provide a clinically oriented, stepwise diagnostic framework for the prenatal evaluation of fetal hepatosplenomegaly, while critically reviewing current evidence on its etiologies, diagnostic limitations, and implications for prenatal and postnatal management.

## 3. Methodology

A literature search was performed using the PubMed/MEDLINE and Cochrane databases, covering publications issued until January 2026. Search terms included combinations of “fetal hepatomegaly”, “fetal splenomegaly”, “fetal hepatosplenomegaly”, “prenatal diagnosis”, “congenital infection”, “fetal anemia”, “lysosomal storage disease”, “genetic disorders”, “fetal therapy”, and “prenatal ultrasound”, combined using Boolean operators (AND/OR). Only English-language manuscripts were considered, with rare exceptions made for historically significant non-English reports without an available English-language equivalent.

Reference lists of all eligible articles were manually screened to identify additional relevant publications not captured by the primary database search. Priority was given to clinical practice guidelines issued by international societies, systematic reviews, meta-analyses, and large cohort studies; case reports and case series were included selectively, restricted to exceptionally rare conditions for which no larger dataset existed.

Given the narrative design of this review, reference selection was performed by the authors based on clinical and academic judgment regarding relevance to the proposed diagnostic algorithm.

It should be noted from the outset that the diagnostic algorithm proposed in this review represents an expert-derived, consensus-based framework, synthesized from the current literature, rather than a validated algorithm confirmed by prospective clinical studies.

## 4. Prenatal Diagnosis of Fetal Hepatosplenomegaly

Routine fetal ultrasound examinations do not include the direct assessment of liver or spleen size [4,5,6]. Consequently, fetal hepatosplenomegaly may remain undetected unless specifically suspected. An enlarged abdominal circumference (AC) may represent the first indirect sonographic sign, although it is neither sensitive nor specific. The visualization of the fetal spleen in the standard AC plane, or the abnormal anterior displacement of the stomach, constitutes another indication for a prompt, dedicated examination of the upper fetal abdomen, including the assessment of liver and spleen size [10,11]. The targeted examination of the fetal liver and spleen is usually performed when hepatosplenomegaly is clinically suspected, such as in cases of unexplained fetal anemia, hydrops fetalis, congenital infection, fetal tumors, metabolic disorders, chromosomal abnormalities, poorly controlled maternal diabetes, or unexplained fetal macrosomia.

At present, no universally accepted prenatal definition of hepatomegaly or splenomegaly exists. The diagnosis relies on biometric measurements exceeding gestational-age-specific reference ranges derived from earlier normative fetal ultrasound studies [12,13,14]. In clinical practice, organ enlargement is generally defined as a measurement above the 95th percentile (or, alternatively, a z-score greater than 2) for gestational age, based on validated reference charts. As measurement techniques and reference populations vary across studies, the same reference chart should be used consistently throughout follow-up examinations.

The accurate biometric assessment of the fetal liver and spleen is subject to considerable technical and biological variability. Fetal position, acoustic shadowing from adjacent structures, difficulty distinguishing organ boundaries from the surrounding tissue, and an increased maternal abdominal wall thickness may all limit image quality and measurement accuracy, particularly in the third trimester. Organ growth is also inherently dynamic across gestation, further complicating single-timepoint interpretation. While linear biometry (length) remains the standard and practically feasible approach in routine prenatal ultrasound, volumetric assessment using three-dimensional ultrasound was proposed as a potentially more reliable alternative [14], though it is not yet standardized or widely adopted in routine clinical practice. Intra- and interobserver variability is a well-documented source of measurement uncertainty in fetal ultrasound biometry [15], and likely applies similarly to liver and spleen measurement, where serial assessment is required. Therefore, testing should be performed by the same examiner using the same reference chart throughout the follow-up.

### 4.1. Fetal Spleen

The fetal spleen is located in the left upper quadrant of the abdomen, beneath the diaphragm and posterolateral to the stomach. It is best visualized in a transverse section through the upper fetal abdomen, at or slightly cranial to the standard abdominal circumference plane [12].

Splenic length is the most reproducible sonographic parameter. It is measured along the longest axis of the organ from the superior to the inferior pole. Some authors additionally measured the maximal transverse diameter or the splenic area. Earlier publications proposed estimating splenic circumference from linear measurements; however, the approach was not widely validated and is not routinely recommended in contemporary fetal ultrasound practice. Therefore, splenic length remains the preferred parameter for prenatal assessment and should be compared against gestational-age-specific normative charts [12,13,14,16,17].

**Figure 1:**
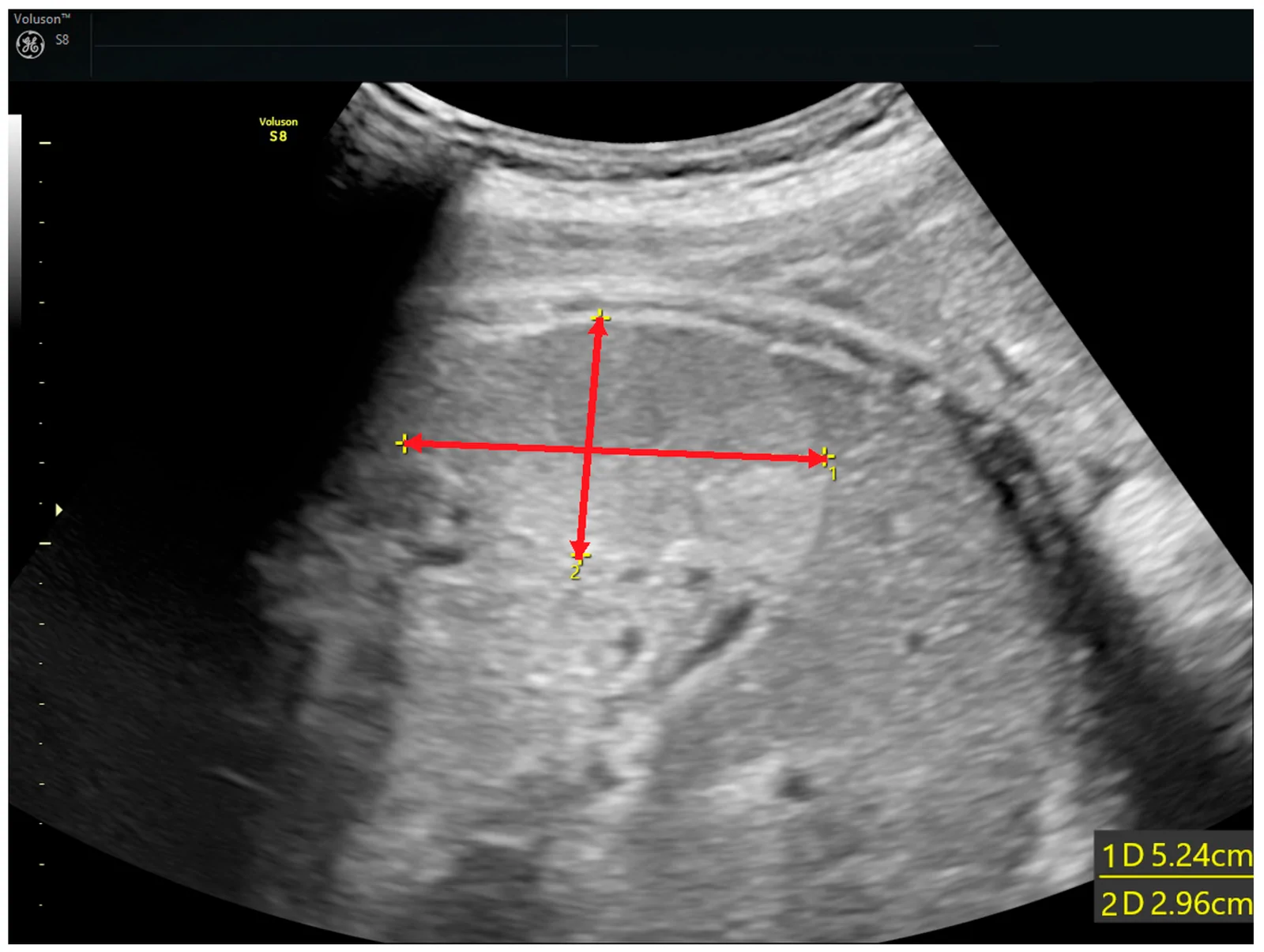
Splenic measurement on transverse abdominal scan in the fetus at 38 gestational weeks (1—splenic length; 2—splenic width).

Several reference charts have been published, all demonstrating a strong linear correlation between splenic dimensions and gestational age [12,13,14,16,17]. Although minor differences exist among these datasets, they provide comparable reference ranges for identifying fetal splenomegaly (Figure 1).

### 4.2. Fetal Liver

The fetal liver is most accurately assessed in a longitudinal sagittal view of the right hepatic lobe. After identifying the descending aorta, the transducer is rotated to obtain a true sagittal section, and liver length is measured from the diaphragmatic dome to the inferior tip of the right lobe. This technique demonstrates good reproducibility and correlates closely with gestational age [10].

**Figure 2:**
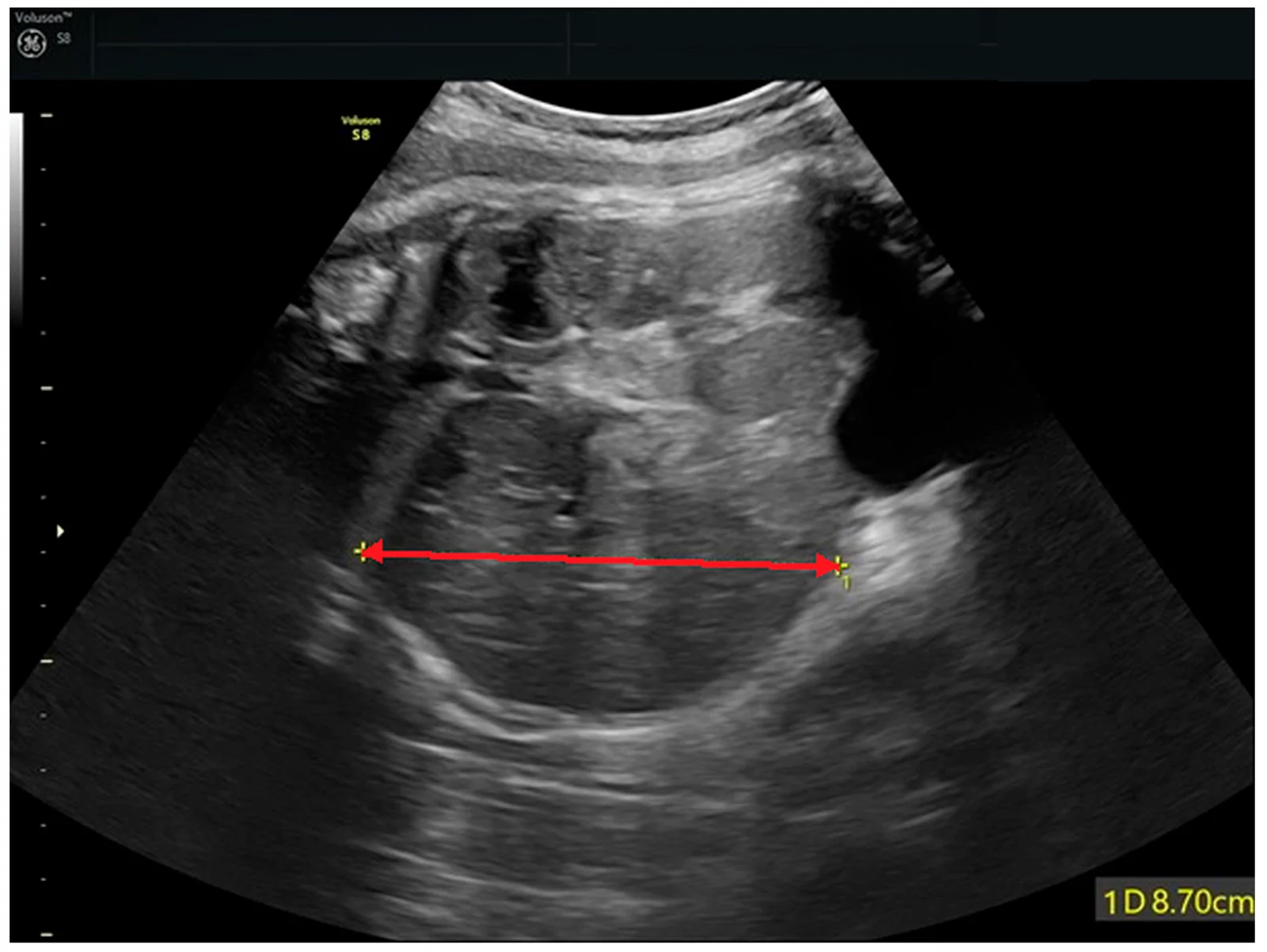
Liver measurement on longitudinal abdominal scan in the fetus at 38 gestational weeks with hepatosplenomegaly due to fetal leukemia (1—liver length).

Reference values established by Vintzileos et al. remain the most commonly cited normative data for fetal liver length [10]. As with splenic assessment, hepatomegaly is diagnosed when liver measurements exceed the 95th percentile or have a z-score greater than +2 for gestational age, provided that technical factors and fetal position allow accurate measurement. Serial assessment using the same reference chart is recommended whenever progressive enlargement is suspected (Figure 2).

Fetal hepatosplenomegaly is diagnosed when both hepatic and splenic measurements exceed gestational-age-adjusted reference limits. Since an isolated, mild enlargement of either organ may occasionally represent a physiological variation, such findings should always be interpreted within the overall clinical context. Clinically, it is useful to distinguish isolated hepatomegaly, isolated splenomegaly, and combined hepatosplenomegaly, as the differential diagnosis differs according to which organ is predominantly affected: several hematological and infectious causes discussed in this review present with prominent splenic involvement, while several metabolic and storage disorders present with prominent hepatic involvement, as detailed in the sections below.

## 5. Diagnostic Protocol

**Figure 3:**
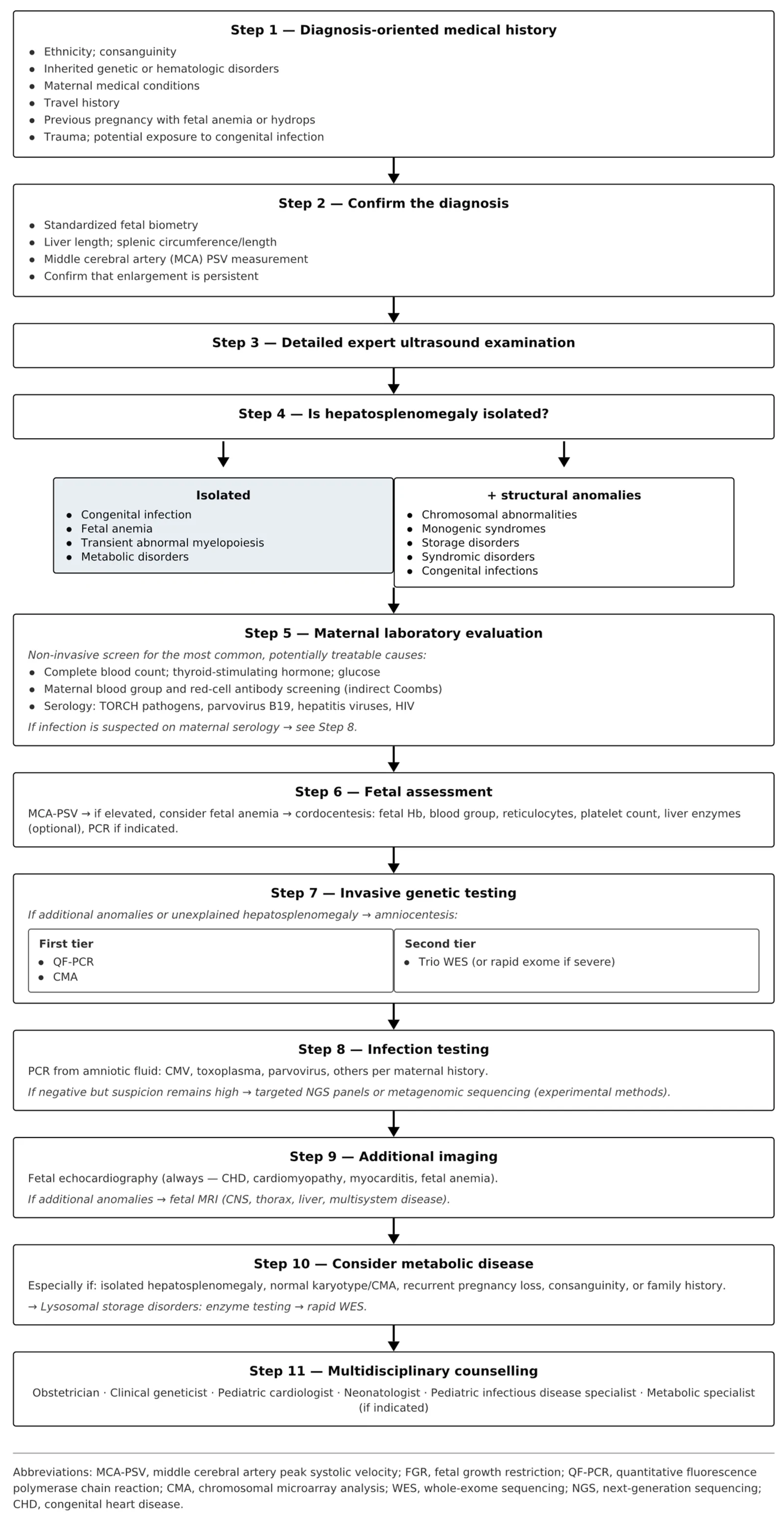
Proposed diagnostic algorithm for fetal hepatosplenomegaly.

For the diagnostic evaluation of fetal hepatosplenomegaly, we propose a stepwise algorithm that prioritizes common and potentially treatable conditions before proceeding to more specialized investigations (Figure 3). Initial assessment should rely on readily available, noninvasive diagnostic methods, with more advanced, invasive, or highly specialized investigations reserved for cases in which first-line testing is inconclusive or suggests a specific underlying disorder.

The first step is the confirmation of true hepatomegaly and/or splenomegaly by detailed prenatal ultrasound. The ultrasound examination should simultaneously assess associated abnormalities, including hydrops fetalis, placental abnormalities, fetal growth abnormalities, structural malformations, signs of congenital infection, and the evidence of hyperkinetic circulation. The Doppler evaluation of the middle cerebral artery peak systolic velocity (MCA-PSV) should be routinely performed to screen for fetal anemia [18].

Concurrently, a detailed maternal and family history should be obtained, focusing on parental ethnicity, consanguinity, inherited genetic or hematologic disorders, maternal medical conditions, travel history, previous pregnancies complicated by fetal anemia or hydrops fetalis, trauma, and potential exposure to congenital infection.

The next stage consists of first-line maternal laboratory investigations aimed at the noninvasive identification of the most common and potentially treatable causes. These include a complete blood count, thyroid-stimulating hormone, glucose assessment, maternal blood group and red cell antibody screening, and serological testing for congenital infections, including TORCH pathogens, parvovirus B19, hepatitis viruses, and HIV. The results of these readily available investigations can provide important clues to the underlying etiology of fetal hepatosplenomegaly, enabling a more focused and efficient diagnostic workup while facilitating the early recognition of treatable conditions, such as fetal anemia or congenital infection [1].

Genetic testing should be considered in every fetus undergoing invasive prenatal diagnosis, regardless of the suspected etiology, as chromosomal and monogenic disorders represent important causes of fetal hepatosplenomegaly [1]. In particular, the presence of structural anomalies or sonographic markers suggestive of chromosomal aberrations constitutes a strong indication for comprehensive genetic testing.

Chromosomal microarray analysis (CMA) should be performed as the first-tier genomic test, as it enables the detection of pathogenic copy number variants that cannot be identified by conventional karyotyping. Although CMA may be performed on either chorionic villi or the amniotic fluid, fetal hepatosplenomegaly is usually diagnosed in the second or third trimester, making amniocentesis the preferred and most commonly used source of fetal DNA for genetic testing [19]. Conventional karyotype analysis remains valuable when numerical or structural chromosomal abnormalities, balanced rearrangements, or mosaicism are suspected [20]. Whenever invasive prenatal testing is performed, sufficient fetal DNA should be stored for potential future analyses. The preservation of genetic material allows additional molecular investigations to be undertaken without the need for repeat invasive procedures.

If CMA is normal and fetal hepatosplenomegaly remains unexplained, particularly in the presence of additional structural abnormalities or a family history suggestive of an inherited disease, next-generation sequencing (NGS)-based testing should be considered. Depending on the clinical phenotype, this may include targeted gene panels for disorders associated with hepatosplenomegaly, such as lysosomal storage disorders, congenital disorders of glycosylation, or hemoglobinopathies. Whole-exome sequencing (WES), preferably performed as trio analysis including parental samples, is recommended in selected cases with multiple congenital anomalies or persistent isolated hepatosplenomegaly after the exclusion of chromosomal abnormalities. Whole-genome sequencing (WGS) may further increase the diagnostic yield in highly selected cases. The choice and sequence of these genetic investigations should be individualized according to the presence of associated structural abnormalities, family history, previous test results, and appropriate genetic counselling, rather than following a fixed algorithm. Genetic counselling is essential to discuss the diagnostic yield, limitations, potential incidental findings, and implications for pregnancy management and recurrence risk [7].

When first-line investigations suggest fetal infection, amniocentesis with pathogen-specific PCR remains the preferred method for confirming congenital infection [3].

Cordocentesis should be performed when moderate or severe fetal anemia is suspected based on MCA-PSV Doppler, or when fetal hematologic disease remains a diagnostic consideration. Besides measuring fetal hemoglobin concentration, cordocentesis enables intrauterine transfusion when indicated [18]. In rare, unexplained cases, fetal blood obtained by cordocentesis may also be evaluated using peripheral blood smear and flow cytometry to exclude fetal leukemia or other hematologic disorders [21].

Because cardiac dysfunction may both cause and result from hepatosplenomegaly, fetal echocardiography should be performed in all affected fetuses [22]. Fetal MRI, although not routinely required, may provide complementary anatomical information in selected cases, particularly when ultrasound findings are inconclusive or when additional central nervous system or abdominal abnormalities are suspected [23].

The proposed algorithm (Figure 3) emphasizes progression from noninvasive assessment to targeted invasive investigations, ensuring that common, treatable etiologies are excluded before extensive testing is undertaken. Management should be coordinated within a multidisciplinary fetal medicine team, integrating maternal–fetal medicine specialists, clinical geneticists, pediatric hematologists, infectious disease specialists, neonatologists, and pediatric cardiologists. Prenatal counseling should address diagnostic uncertainty, available evidence-based prenatal therapies, delivery planning, the need for targeted postnatal investigations, and long-term follow-up.

The following sections review the principal etiologies of fetal hepatosplenomegaly, outlining the underlying pathophysiological mechanisms together with the relevant diagnostic workup and potential therapeutic strategies to facilitate clinical decision-making in individual cases (Figure 3).

## 6. Possible Etiologies of Fetal Hepatosplenomegaly

### 6.1. Congenital Infections

Although fetal hepatosplenomegaly is not a typical or common manifestation of congenital infection, the relatively high prevalence of congenital infectious diseases and the availability of noninvasive maternal serological testing make the exclusion of fetal infection one of the first diagnostic steps in the evaluation of a fetus presenting with hepatosplenomegaly. The detailed spectrum of infectious agents and symptoms is summarized in Table 1.

### 6.2. Cytomegalovirus (CMV) Infection

Although various structural abnormalities may be detected antenatally in CMV-affected fetuses, hepatosplenomegaly is reported only in a minority of cases [24,25]. On fetal MRI, hepatosplenomegaly and abnormal hepatic signal intensity may represent nonspecific findings. However, their coexistence with additional abnormalities should raise a suspicion of fetal infection [26].

When present, hepatosplenomegaly may result from cholestatic hepatitis, liver dysfunction, and extramedullary hematopoiesis. Associated ultrasound findings may include echogenic foci corresponding to calcifications, microabscesses, or focal necrosis. However, sonographic features are nonspecific and overlap with other TORCH (Toxoplasmosis, Other, Rubella, Cytomegalovirus, Herpes) infections. Therefore, the diagnosis relies on maternal serology and confirmation by amniotic fluid PCR [1].

Although CMV is the most common congenital infection, most infected fetuses remain asymptomatic. Nevertheless, given its high population prevalence, CMV should be considered in the differential diagnosis of fetal hepatosplenomegaly [1].

In confirmed maternal infection, valaciclovir has emerged as a potential prenatal therapeutic option. Recent European recommendations (European Congenital Cytomegalovirus Initiative; ECCI) support oral high-dose valaciclovir (8 g/day) after primary maternal CMV infection acquired in the periconceptional period or first trimester to reduce vertical transmission (Grade A) [27,28]. In contrast, the ISUOG (International Society of Ultrasound in Obstetrics and Gynecology) and SMFM (Society for Maternal–Fetal Medicine) continue to regard antenatal antiviral therapy for established fetal CMV infection as investigational and do not recommend its routine use outside specialized centers or research protocols [3,27,29]. Available data suggest a possible reduction in congenital disease manifestations with an acceptable safety profile. However, this approach remains investigational and requires further validation [28].

### 6.3. Toxoplasmosis

Hepatosplenomegaly represents the most common extracerebral manifestation of congenital toxoplasmosis. In fetuses with PCR-confirmed infection and abnormal ultrasound findings, it was reported in 17% of cases [30].

The pathogenesis involves both inflammatory immune activation and direct parasitic invasion. Fetal immune response to *Toxoplasma gondii* leads to hepatic and splenic inflammation, tissue injury, and organ enlargement [31,32]. The direct invasion of hepatocytes and splenocytes, together with the activation of extramedullary hematopoiesis secondary to systemic inflammation and anemia, further contributes to hepatosplenomegaly [33,34].

The diagnosis is based on maternal serology and confirmed by amniotic fluid PCR [35]. In confirmed maternal infection, spiramycin is administered to reduce vertical transmission, although it does not treat an established fetal infection. When fetal transmission is confirmed after 14 weeks of gestation, combined pyrimethamine and sulfadiazine therapy is recommended to decrease the risk of symptomatic congenital disease [35].

### 6.4. Congenital Syphilis

Hepatosplenomegaly is a frequent and early manifestation of congenital syphilis, resulting from a combination of direct invasion by the spirochete *Treponema pallidum* and immune-mediated inflammation, including syphilitic hepatitis, extramedullary hematopoiesis secondary to anemia, and venous congestion due to cardiac dysfunction [36].

Fetal manifestations typically evolve in a characteristic sequence, with hepatomegaly and placentomegaly representing early signs, followed by hematologic abnormalities, central nervous system involvement, and ascites [37].

Clinical presentation is gestational-age-dependent. In early pregnancy, infection may remain histopathologically detectable but sonographically silent, reflecting the immaturity of the fetal immune response. Multiorgan inflammatory manifestations, including hepatosplenomegaly, correlate with immune system maturation around 18 weeks of gestation [37,38].

Maternal screening is based on serological testing, while definitive diagnosis may rely on darkfield microscopy or molecular testing (PCR) from lesion swabs [38]. Following adequate penicillin therapy, fetal manifestations typically resolve in a reverse order, with hydrops fetalis or ascites regressing before hepatomegaly and placentomegaly [37].

### 6.5. Parvovirus B19

Hepatosplenomegaly in parvovirus B19 infection is primarily secondary to severe fetal anemia, which may progress to hydrops fetalis, cardiac failure, and intrauterine death. The virus selectively infects erythroid precursor cells, leading to apoptosis and aplastic anemia. Direct hepatic involvement may further contribute to hepatomegaly [39].

Maternal infection is usually mild and nonspecific, and seroconversion during pregnancy occurs in approximately 1–5% of cases. The diagnosis is based on maternal serology, with the confirmation of fetal transmission by PCR on amniotic fluid [40].

Once fetal infection is suspected, close surveillance for anemia is required using middle cerebral artery peak systolic velocity (MCA-PSV) Doppler assessment [40]. In cases of severe anemia, cordocentesis with intrauterine blood transfusion represents an effective treatment and may result in full recovery [40].

### 6.6. Fetal Anemia

Severe fetal anemia may result in hepatosplenomegaly due to compensatory extramedullary hematopoiesis in the fetal liver and spleen. Regardless of etiology, screening is performed using MCA-PSV Doppler assessment, and the diagnosis is confirmed by cordocentesis with fetal blood sampling. Untreated severe anemia may progress to high-output cardiac failure, hydrops fetalis, and intrauterine death [18].

Etiologies include congenital infections (e.g., CMV, parvovirus B19), hemoglobinopathies, and Rhesus disease or other forms of hemolytic disease of the fetus and newborn (HDFN) [18].

HDFN results from the transplacental passage of maternal alloantibodies directed against fetal red blood cells. While anti-RhD immunization has markedly declined due to routine prophylaxis, anti-E and anti-K antibodies are currently among the most clinically significant forms [18]. Cell-free fetal DNA testing enables noninvasive fetal antigen determination and may guide targeted anti-D immunoglobulin administration [41].

Acquired bone marrow dysfunction, such as aplastic anemia, may be induced by toxins or radiation, although this is extremely rare in fetuses [18]. Drug toxicity may also cause fetal anemia, including chemotherapy agents that induce bone marrow suppression (cytarabine, ifosfamide, cisplatin, and doxorubicin) [42,43] and mesalazine [44].

In severe cases, intrauterine transfusion (IUT) remains the standard therapeutic intervention, significantly improving perinatal survival and long-term outcomes. However, repeated transfusions may lead to iron overload and secondary hepatosplenomegaly [45].

Fetal anemia may also arise in monochorionic pregnancies with placental vascular anastomoses, particularly in twin anemia-polycythemia sequence (TAPS) or twin reversed arterial perfusion (TRAP) sequence [18].

### 6.7. Congenital Hemolytic Anemia

Congenital hemolytic anemias are a heterogeneous group of diseases that share hepatosplenomegaly as a common pathological feature. Increased erythrocyte breakdown leads to red blood cell deficiency, which stimulates extramedullary hematopoiesis in the fetal liver and spleen, resulting in organ enlargement. Hemosiderin deposition, portal hypertension, and hepatic vascular congestion may further contribute to this enlargement [46].

### 6.8. Cell Membrane Defects: Spherocytosis

Spherocytosis is the most common congenital hemolytic anemia (1:5000 live births), usually inherited in an autosomal dominant manner. Mutations affecting spectrin or ankyrin disrupt the erythrocyte membrane skeleton, leading to the loss of membrane stability. Red blood cells become spherical, less deformable, and prone to premature destruction. Approximately 5% of cases are severe and may present prenatally with fetal anemia, hydrops fetalis, or stillbirth [47].

Parental testing often confirms the mutation. In suspected *de novo* or autosomal recessive cases, molecular testing (Sanger sequencing or NGS) may be performed on amniotic fluid. Cordocentesis allows the confirmation of fetal anemia. In severe cases, intrauterine transfusion may resolve life-threatening hydrops fetalis [47].

### 6.9. Hemoglobinopathies

Liver and spleen enlargement in hemoglobinopathies is more pronounced than in other hemolytic anemias. This results from the massive breakdown of abnormal hemoglobin within the liver. Hepatic clearance mechanisms become overwhelmed, leading to the accumulation of hemoglobin degradation products within the small intrahepatic vessels. This results in vascular congestion and impaired hepatic blood flow. The ensuing hepatic stasis places additional hemodynamic strain on the fetal heart, which is already compromised by severe anemia [46].

### 6.10. Glucose-6-Phosphate Dehydrogenase Deficiency

This enzyme deficiency causes transient hemolysis triggered by oxidative stressors such as sulfonamide exposure or fava bean ingestion. Only five cases of severe prenatal anemia with hydrops fetalis have been reported to date [48].

### 6.11. Thalassemia

Thalassemias are inherited hemoglobinopathies caused by the reduced synthesis of alpha- or beta-globin chains. The most severe form, alpha-thalassemia major (Hb Bart’s disease), presents prenatally with profound anemia, hypoxia, hydrops fetalis, and high perinatal mortality. Absent alpha-chain production leads to the formation of gamma4 tetramers (Hb Bart’s), which deliver oxygen ineffectively [49].

Severe intrauterine hypoxia may impair organogenesis, with structural anomalies being reported in approximately 20% of affected fetuses [50].

Screening is recommended in women with microcytic anemia and normal ferritin levels, using parental hemoglobin electrophoresis as first-line assessment [51]. In at-risk pregnancies, chorionic villus sampling (CVS) or amniocentesis confirms the diagnosis, while cordocentesis with fetal hemoglobin electrophoresis may provide definitive evaluation. Noninvasive prenatal testing (NIPT) was also developed for alpha-thalassemia screening [52].

In alpha-thalassemia major, intrauterine transfusions (IUT) initiated from around 18 weeks significantly improve survival; repeated IUTs may enable survival to delivery and access to postnatal therapies, including stem cell transplantation [50].

A recent CLIMB THAL-111 gene therapy trial revealed transfusion independence in 91% of patients with beta-thalassemia treated with exagamglogene autotemcel [53].

### 6.12. Congenital Sideroblastic Anemia with B-Cell Immunodeficiency, Periodic Fevers, and Developmental Delay

Congenital sideroblastic anemia with B-cell immunodeficiency, periodic fevers, and developmental delay (SIFD) is a rare *TRNT1*-related disorder characterized by microcytic anemia, immunodeficiency, recurrent fevers, and neurodevelopmental impairment [54,55].

Hepatosplenomegaly is attributed to iron overload and extramedullary hematopoiesis [56].

No established prenatal therapy exists. Isolated reports described a response to etanercept, while bone marrow transplantation and thalidomide were considered in postnatal management [57].

### 6.13. Hepatosplenomegaly in Genetic Disorders

Hepatosplenomegaly may be a prenatal manifestation of numerous genetic abnormalities, including simple trisomies as well as rare monogenic disorders, as shown in Table 2.

### 6.14. Trisomy 21

Trisomy 21 is the most common chromosomal disorder and is frequently associated with transient abnormal myelopoiesis (TAM), a preleukemic condition characterized by the proliferation of circulating megakaryoblasts. Hepatosplenomegaly results from extramedullary hematopoiesis, megakaryoblastic infiltration, and progressive liver fibrosis [58]. TAM occurs in approximately 10% of individuals with trisomy 21 [59]. Hepatosplenomegaly is a prominent ultrasound finding and may represent the first abnormal sign leading to the diagnosis. The presence of hydrops fetalis indicates a poor prognosis [60].

Pathogenesis involves trisomy-21-related gene dosage effects and a concomitant *GATA1* mutation, which together drive abnormal megakaryocyte proliferation [61,62]. Clinical presentation ranges from spontaneous postnatal resolution to severe hydrops fetalis and liver failure [60]. Prenatal diagnosis requires the confirmation of trisomy 21 and fetal blood analysis via cordocentesis demonstrating circulating blasts and cytopenias. The detection of a *GATA1* mutation supports the diagnosis [60,61]. Supportive prenatal management with intrauterine transfusion was reported in isolated cases; however, evidence is insufficient to recommend routine prenatal systemic therapy [60].

Hepatosplenomegaly may also occur in fetuses with trisomy 21 in rare cases of congenital acute myeloid leukemia [63].

### 6.15. Beckwith–Wiedemann Syndrome (BWS) and Other Rare Overgrowth Syndromes

Beckwith–Wiedemann syndrome (BWS) is the most frequent overgrowth syndrome, in which hepatosplenomegaly is caused by genetically determined organomegaly [64]. The syndrome results from genetic or epigenetic alterations of the imprinted 11p15 region, leading to dysregulated growth control, including abnormal *IGF2* expression [65]. Molecular mechanisms include methylation abnormalities, copy number variants, and *CDKN1C* mutations. The diagnosis requires targeted molecular testing (methylation analysis, sequencing, or CMA). A positive family history is present in approximately 15% of cases [66].

Common prenatal features include macrosomia, visceromegaly, macroglossia, omphalocele, adrenal and renal abnormalities, polyhydramnios, and placental mesenchymal dysplasia (PMD) [65,67].

There is no specific prenatal therapy. Careful obstetric surveillance is recommended, particularly after 22 weeks of gestation, given the increased risk of developing gestational diabetes, hypertensive disorders, and perinatal complications related to macrosomia [68].

Hepatosplenomegaly may also occur in other overgrowth syndromes, including Sotos, Weaver, Perlman, and Malan syndromes [69].

### 6.16. Lysosomal Storage Diseases

Lysosomal storage diseases (LSDs) are a heterogeneous group of nearly 70 disorders caused by lysosomal enzyme deficiencies resulting from genetic mutations. Excessive substrate accumulation impairs lysosomal function in multiple tissues. Although most affected fetuses do not present specific sonographic signs, LSDs account for approximately 15% of cases of non-immune hydrops fetalis (NIHF) [70]. Hepatosplenomegaly results from substrate accumulation, hepatic fibrosis, cirrhosis, ascites, and hydrops fetalis [1,70,71].

On ultrasound, hepatosplenomegaly may be associated with the characteristic “starry sky” appearance of the liver, resulting from the fibrous expansion of the portal spaces, and a reticulonodular surface reflecting cirrhosis. The assessment of the fetal liver surface is possible in the presence of ascites [1], which may result from liver insufficiency and anemia secondary to hypersplenism [70].

Excessive storage may lead to a broad spectrum of signs and symptoms manifesting from the prenatal period through adulthood [72].

Prenatal diagnosis is usually confirmed by CMA, NGS, or Sanger sequencing of fetal material obtained by CVS or amniocentesis, while biochemical enzyme assays remain the diagnostic gold standard in selected cases [73]. Tandem mass spectrometry of amniotic fluid, measuring disease-specific oligosaccharide and glycolipid profiles, was first demonstrated as a means of in utero detection of lysosomal storage disorders in affected pregnancies [74], and remains a recognized complementary biochemical approach to enzyme-based diagnosis [75].

In recent years, several therapeutic strategies have been investigated to improve quality of life in patients with LSDs. Enzyme replacement therapy (ERT)—a specific, targeted treatment based on the fact that LSDs are caused by an enzyme deficiency—remains experimental. The regular administration of recombinant enzymes allows lysosomes to maintain their normal metabolic function. Such treatment may prevent the consequences of the enzymatic block. However, it does not reverse damage that has already occurred [76].

In utero enzyme replacement therapy (IUERT) with recombinant enzymes may overcome some of the limitations seen with postnatal therapies. As gene therapies continue to develop, IUERT may, in the future, become a bridge that allows individuals with severe phenotypes to benefit from definitive therapies after birth [76].

### 6.17. Rare Causes of Hepatosplenomegaly

#### 6.17.1. Arteriovenous Shunts Resulting in Hyperkinetic Circulation

In conditions associated with the sequestration or destruction of fetal red blood cells, fetal anemia may develop. This is observed in highly vascular tumors such as sacrococcygeal teratoma (SCT), placental chorioangioma, and fetal hemangiomas [18]. High-output cardiac failure may ensue, resulting in systemic venous congestion and fluid accumulation. Secondary hepatic and splenic congestion manifests as hepatosplenomegaly and may contribute to hydrops fetalis [77].

#### 6.17.2. Diabetic Fetopathy

Fetal hepatosplenomegaly may be detected in diabetic fetopathy [77,78,79]. As a consequence of maternal hyperglycemia, fetal hypoxia is often observed. Fetal compensatory mechanisms, mediated by erythropoietin (EPO), include increased extramedullary erythropoiesis in the liver and spleen, leading to their enlargement [78]. In some cases, excessive hematopoiesis may result in fetal polycythemia and cause vascular congestion, particularly in the liver and spleen, contributing to their enlargement [79]. Insulin is an anabolic hormone. Therefore, in the presence of fetal hyperinsulinemia, organomegaly—including hepatomegaly—is commonly observed [77].

#### 6.17.3. Acute Fetal Leukemia

Acute fetal leukemia should be considered in cases of hepatosplenomegaly concomitant with hydrops fetalis, after the exclusion of infectious, immune, and metabolic causes. It is particularly associated with genetic syndromes such as Down syndrome, Bloom syndrome, Noonan syndrome, ataxia–telangiectasia, Wiskott–Aldrich syndrome, and Li–Fraumeni syndrome. Hepatosplenomegaly is the predominant prenatal finding and may coexist with anemia, fetal growth restriction (FGR), placentomegaly, and thymic hypoplasia [21].

The enlargement results from leukemic infiltration and extramedullary hematopoiesis. The diagnosis is established by fetal blood smear and flow cytometry, with genetic abnormalities present in over 50% of cases. In Down syndrome, approximately 10% of TAM cases progress to leukemia [21].

#### 6.17.4. Congenital Thyrotoxicosis

In fetuses of mothers with active Graves’ disease, hepatosplenomegaly may develop due to transplacental thyroid-stimulating antibodies, including TRAb and TSI (thyrotropin receptor antibodies and thyroid-stimulating immunoglobulins) [80]. Severe cases may lead to fetal heart failure and arrhythmias with secondary hepatic and splenic congestion, whereas milder forms are associated with hyperkinetic circulation and increased organ perfusion. Additional contributing mechanisms include extramedullary hematopoiesis secondary to mild anemia or thrombocytopenia, and immune system activation [81].

#### 6.17.5. Primary Hemophagocytic Lymphohistiocytosis (HLH)

Primary HLH is a rare, autosomal recessive hyperinflammatory disorder caused by an impaired cytotoxic function of NK and T cells. Prenatal hepatosplenomegaly was reported in affected fetuses [2].

#### 6.17.6. Langerhans Cell Histiocytosis (LCH)

Fetal LCH is an exceptionally rare clonal proliferative disorder that may involve the liver and spleen, presenting with hepatosplenomegaly, cytopenias and, occasionally, hydrops fetalis [82,83].

## 7. Emerging Diagnostic and Therapeutic Advances

Recent technological developments continue to reshape the prenatal evaluation of fetal hepatosplenomegaly. In the field of congenital infections, metagenomic next-generation sequencing is an emerging, not yet routinely implemented approach expanding the range of detectable pathogens beyond targeted RT-PCR panels [84], and microbial cell-free DNA sequencing has recently been proposed as an investigational adjunct to conventional diagnostics in neonatal sepsis, a concept that may extend to amniotic fluid analysis in suspected congenital infection, though clinical validation in this setting is still lacking [85].

Genetic and monogenic disorder diagnostics have advanced particularly rapidly, although most remain at the investigational or early-validation stage. Noninvasive fetal exome sequencing from a maternal blood sample, rather than from invasively obtained fetal material, has recently been demonstrated as technically feasible in proof-of-concept studies, but is not yet part of established clinical practice [86,87], while cell-free DNA screening is being extended from aneuploidy detection toward dominant monogenic conditions on an investigational basis [88]. A combined noninvasive detection of both chromosomal and monogenic abnormalities within a single assay was also proposed as an experimental approach [89]. Optical genome mapping, long-read sequencing, and RNA sequencing remain investigational but may further refine variant interpretation in the coming years. As genomic testing becomes increasingly comprehensive, the interpretation and disclosure of Variants of Uncertain Significance (VUS) represents a growing and explicitly recognized challenge in prenatal genetic counseling. Time pressure and limited phenotypic information make VUS interpretation more difficult in the prenatal setting than postnatally. Moreover, there is active debate regarding whether and how such variants should be reported to parents, given their potential to influence pregnancy decision-making despite their unresolved clinical significance [90].

The imaging of the fetal liver and spleen is similarly evolving beyond conventional biometry. Fetal MRI can now provide quantitative signal-intensity and volumetric data reflecting organ iron content and disease burden, as demonstrated in fetuses undergoing intrauterine transfusion, an application increasingly used in specialized centers, though not yet standardized [45]. Elastography, already established for postnatal liver fibrosis assessment but not yet validated prenatally, and artificial-intelligence-assisted automated organ segmentation and volumetry—increasingly applied in fetal brain imaging—represent promising, though still investigational, directions for a more objective and reproducible assessment of fetal hepatosplenomegaly.

Magnetic resonance spectroscopy was applied, in an investigational capacity, in fetal CMV infection to characterize brain tissue changes (e.g., myo-inositol peaks reflecting white matter involvement) in cases with concurrent hepatosplenomegaly on conventional imaging [26]. Spectroscopic characterization of the liver itself has not yet been reported in the fetal congenital infection literature, representing a potential direction for future research.

Beyond diagnostics, prenatal therapeutic options continue to expand, though the level of clinical validation differs substantially across approaches. Intrauterine transfusion for fetal anemia is an established, guideline-supported intervention, whereas in utero enzyme replacement therapy for lysosomal storage disorders remains experimental, currently restricted to highly selected cases and research protocols. These are increasingly complemented by even earlier-stage emerging approaches, including in utero stem cell transplantation and gene therapy, which may eventually extend curative treatment to a broader range of monogenic and metabolic conditions underlying fetal hepatosplenomegaly [9]. In utero hematopoietic stem cell transplantation has achieved clinical success in an individual reported case of X-linked severe combined immunodeficiency, illustrating the therapeutic potential of this approach without yet constituting established clinical practice for other hematologic and metabolic disorders [91]. Gene therapy for prenatal correction remains at a preclinical/early-investigational stage; continued refinement of viral and non-viral gene-delivery platforms is expected to broaden the range of conditions amenable to prenatal correction in the future.

## 8. Discussion

Fetal hepatosplenomegaly represents a manifestation of numerous disorders rather than a diagnosis itself [1]. Its heterogeneous etiology frequently results in extensive but non-standardized investigations and creates considerable challenges for prenatal counseling, clinical decision-making, and postnatal care planning. Therefore, the principal objective of this review was not only to summarize the etiological spectrum of fetal hepatosplenomegaly but also to translate the available evidence into a practical, stepwise diagnostic framework that may facilitate everyday clinical practice.

A central message emerging from this review is that diagnostic evaluation should proceed from readily available, noninvasive investigations toward increasingly specialized and invasive testing. Detailed fetal ultrasound remains the cornerstone of diagnosis, as it confirms true hepatomegaly and/or splenomegaly while simultaneously identifying associated abnormalities that substantially narrow the differential diagnosis.

Another important observation is that advances in prenatal molecular diagnostics have fundamentally changed the evaluation of unexplained fetal hepatosplenomegaly. Chromosomal microarray analysis has become the first-tier genomic investigation whenever invasive prenatal testing is indicated, whereas next-generation sequencing—particularly trio whole-exome sequencing—has substantially increased diagnostic yield in fetuses with additional structural abnormalities or persistent isolated organomegaly after the exclusion of chromosomal abnormalities [7]. As fetal hepatosplenomegaly is usually detected during the second or third trimester, amniocentesis rather than chorionic villus sampling is the predominant source of fetal DNA. Furthermore, the preservation of fetal DNA obtained during invasive procedures should be considered whenever possible, allowing future reanalysis as novel disease-associated genes are identified [19].

This review also highlights the importance of prioritizing potentially treatable etiologies early in the diagnostic pathway. Congenital infections and fetal anemia account for a substantial proportion of prenatally diagnosed cases [1,2] and may benefit from timely prenatal interventions, including maternal antimicrobial therapy [28,35], intrauterine transfusion [18], or optimized perinatal management [3]. Conversely, for many genetic and metabolic disorders, prenatal diagnosis primarily informs prognosis, delivery planning, recurrence risk assessment, and preparation for specialized neonatal care [7,50,76]. Emerging prenatal therapies, including in utero enzyme replacement therapy, represent promising developments, but currently remain limited to highly selected conditions and investigational settings. Their role should therefore be interpreted cautiously until supported by larger prospective studies [9,76].

Despite substantial advances in imaging and genomic technologies, important limitations remain, including the lack of universally accepted prenatal definitions of hepatomegaly and splenomegaly and the absence of universal reference charts [1,12]. A mild, isolated enlargement of either organ may occasionally represent a physiological variation, emphasizing that sonographic findings should always be interpreted within the broader clinical context.

This review has several limitations. First, as a narrative rather than systematic review, reference selection was based on the authors’ clinical and academic judgment rather than a prespecified, reproducible protocol, which may introduce selection bias favoring studies that support the proposed diagnostic framework. Second, evidence for several rare etiologies discussed in this review relies predominantly on case reports and small case series, which carry an inherent risk of publication and reporting bias and limit the strength of conclusions that can be drawn regarding their true prevalence and clinical significance. Finally, the proposed diagnostic algorithm has not undergone prospective validation in a clinical cohort; its performance, feasibility, and impact on diagnostic yield and patient outcomes remain to be formally assessed before it can be recommended for routine clinical implementation.

Several unresolved questions continue to limit evidence-based management. The natural history and prognostic significance of isolated fetal hepatosplenomegaly remain poorly defined, particularly in the absence of additional structural abnormalities. Similarly, evidence supporting advanced imaging modalities, including fetal MRI, elastography, radiomics, and functional imaging techniques, remains limited. Further studies are also required to determine the diagnostic value of emerging molecular technologies, including genome sequencing, transcriptomics, metabolomics, and metagenomic approaches for congenital infections [84]. Prospective, multicenter registries will be essential to establish standardized diagnostic criteria, validate proposed algorithms, and better define genotype–phenotype correlations and long-term outcomes.

Finally, prenatal management should extend beyond establishing an etiological diagnosis. Because fetal hepatosplenomegaly may reflect a complex multisystem disease, the management requires close collaboration between maternal–fetal medicine specialists, clinical geneticists, pediatric hematologists, infectious disease specialists, pediatric cardiologists, radiologists, and neonatologists. Multidisciplinary counseling should integrate diagnostic uncertainty, available prenatal therapeutic options, anticipated neonatal management, recurrence risk, and the need for targeted postnatal investigations and long-term follow-up.

In conclusion, fetal hepatosplenomegaly should be regarded as a clinically important prenatal sign requiring a structured, etiology-driven diagnostic approach rather than as an isolated ultrasound finding. Future research should focus on developing standardized diagnostic criteria, validating diagnostic pathways in prospective cohorts, and evaluating the impact of rapidly evolving prenatal genomic and fetal therapeutic technologies on clinical outcomes.

## Figures and Tables

**Table 1:** Possible infectious etiologies of fetal hepatosplenomegaly.

Infection	Ultrasonographic Findings	Diagnostic Methods
CMV infection	**CNS findings:** – Ventriculomegaly – Periventricular hyperechogenicity – Hydrocephalus – Microcephaly – Vermian hypoplasia – Intraventricular adhesions – Intracerebral calcifications **Other findings:** – Fetal growth restriction (FGR) – Hydrops fetalis – Hyperechogenic bowel – Placentomegaly – Oligohydramnios or polyhydramnios	– Maternal serology – RT-PCR from amniotic fluid
Toxoplasmosis	**CNS findings:** – Hyperechogenic nodular foci – Ventriculomegaly – Periventricular hyperechogenicity – Hydrocephalus – Combined central nervous system malformations **Other findings:** – Ascites – Hyperechogenic bowel	– Maternal serology – RT-PCR from amniotic fluid
Congenital syphilis	**Findings:** – Fetal anemia – Ascites – Hydrops fetalis – Pericardial effusion – Cardiomegaly – Placentomegaly – Oligohydramnios or polyhydramnios	– Maternal serology—non-treponemal tests (RPR, VDRL, or TRUST) or treponemal tests (FTA-ABS, TP-EIA, MHA-TP, TPPA, or CIA) – Darkfield microscopy or molecular testing (RT-PCR)
Parvovirus B19	**CNS findings:** – Hydrocephalus – Cerebral hemorrhage – Polymicrogyria **Other findings:** – Fetal anemia – FGR – Hydrops fetalis – Hyperechogenic bowel – Liver calcifications – Cardiac defects (VSD, Ebstein’s anomaly, cardiac block)	– Maternal serology – RT-PCR from amniotic fluid
Rubella	**Findings:** – FGR – Ascites – Cardiomegaly – Atrial septal defect – Hyperechogenic bowel – Placentomegaly – Polyhydramnios	– Maternal serology – RT-PCR from amniotic fluid
Congenital tuberculosis	**Findings:** – – FGR – Hydrops fetalis	– Maternal interferon-gamma release assay (IGRA)
Listeriosis	**CNS findings:** – Intracerebral calcifications **Other findings:** – Hydrops fetalis – Ascites – Hyperechogenic, dilated bowel – Dilated gallbladder	– Maternal serology – RT-PCR from amniotic fluid
Lymphocytic choriomeningitis virus (LCMV)	**CNS findings:** – Ventriculomegaly – Periventricular calcifications – Hydrocephalus – Microcephaly **Other findings:** – Fetal anemia – FGR – Hydrops fetalis – Ascites – Cardiomegaly	– Maternal serology – RT-PCR from amniotic fluid
Typhus	**Findings:** – FGR	– Maternal serology – RT-PCR from amniotic fluid
Enterovirus	**CNS findings:** – Hydrocephalus **Other findings:** – Ascites – Liver calcifications – Placentomegaly	– Maternal serology – RT-PCR from amniotic fluid – Microbiological assessment of amniotic fluid
Zika virus infection	**CNS findings:** – Microcephaly – Ventriculomegaly – Corpus callosum abnormalities – Periventricular calcifications **Other findings:** – Thymic hypertrophy – FGR	– Maternal serology – RT-PCR from amniotic fluid – Zika virus nucleic acid amplification testing (NAAT)

FGR, fetal growth restriction; RT-PCR, reverse transcription polymerase chain reaction; RPR, Rapid Plasma Reagin; VDRL, Venereal Disease Research Laboratory test; TRUST, Toluidine Red Unheated Serum Test; FTA-ABS, fluorescent treponemal antibody absorption; TP-EIA, Treponema pallidum enzyme immunoassay; MHA-TP, microhemagglutination assay for antibodies to Treponema pallidum; TPPA, Treponema pallidum particle agglutination assay; CIA, chemiluminescence immunoassay; VSD, ventricular septal defect; IGRA, interferon-gamma release assay; NAAT, nucleic acid amplification testing.

**Table 2:** Genetic causes of fetal hepatosplenomegaly.

Genetic Disease	Ultrasonographic Findings	Diagnosis
Down syndrome (trisomy 21)	**First-trimester findings:** – Increased nuchal translucency (NT) – Absent or hypoplastic nasal bone (NB) – Abnormal ductus venosus (DV) flow – Tricuspid regurgitation (TR) **Second- and third-trimester findings:** – Increased nuchal fold (NF) – Short long bones – Aberrant right subclavian artery (ARSA) – Ventriculomegaly – Echogenic intracardiac focus – Echogenic bowel – Mild hydronephrosis – Cardiac septal defects – Duodenal atresia – Hydrops fetalis – Macroglossia – Hypoplastic maxilla – Vascular malformations	– Invasive genetic testing with fetal karyotyping – Noninvasive prenatal testing (NIPT) available
Beckwith–Wiedemann syndrome (BWS)	**Findings:** – Macrosomia – Hemihyperplasia – Nephromegaly – Omphalocele – Adrenal hyperplasia – Renal anomalies – Macroglossia – Ear malformations – Polyhydramnios – Placental mesenchymal dysplasia (PMD)	– Methylation-specific multiplex ligation-dependent probe amplification (MS-MLPA)
Noonan syndrome	**Findings:** – Increased nuchal translucency – Hypertrophic cardiomyopathy – Ventricular hypertrophy – Pulmonary stenosis – Absent ductus venosus (DV) flow – Thickened nuchal fold (NF) – Cystic hygroma – Non-immune hydrops fetalis (NIHF) – Polyhydramnios – Ascites	– Invasive genetic testing with targeted sequencing of RAS/MAPK pathway genes: – *PTPN11* (~50%) – *SOS1* (~10%) – *RAF1* (~10%) – *KRAS* (<2%) – *NRAS* (<1%)
Congenital hemolytic anemia—spherocytosis	**Findings:** – Non-immune hydrops fetalis (NIHF)	– Parental carrier testing – Invasive genetic testing with next-generation sequencing (NGS) – Cordocentesis
Congenital hemolytic anemia—G6PD deficiency	**Findings:** – Non-immune hydrops fetalis (NIHF)	– Family history – Cordocentesis for G6PD enzyme assay – Invasive genetic testing with next-generation sequencing (NGS)
Thalassemia	**Findings:** – Microcephaly – Hydrocephalus – Cardiac defects – CNS anomalies – Hypospadias – Limb malformations – Anomalous genitalia – Lung hypoplasia	– Parental hemoglobin (Hb) electrophoresis – Invasive genetic testing with next-generation sequencing (NGS) – Cord blood electrophoresis via cordocentesis – NIPT for α- or β-thalassemia
Lysosomal storage diseases (LSDs)	**Findings:** – Non-immune hydrops fetalis (NIHF) – FGR – CNS anomalies – Echogenic kidneys	– Family history – Parental testing – Amniocentesis with enzymatic and genetic analysis – Invasive genetic testing with next-generation sequencing (NGS)
Congenital sideroblastic anemia with B-cell immunodeficiency, periodic fevers, and developmental delay (SIFD)	**Findings:** – Non-immune hydrops fetalis (NIHF) – Fetal anemia – FGR – Lung hypoplasia – Cardiomegaly	– Invasive genetic testing with next-generation sequencing (NGS)

NT, nuchal translucency; NB, nasal bone; DV, ductus venosus; TR, tricuspid regurgitation; NF, nuchal fold; ARSA, aberrant right subclavian artery; NIPT, noninvasive prenatal testing; NIHF, non-immune hydrops fetalis; PMD, placental mesenchymal dysplasia; MS-MLPA, methylation-specific multiplex ligation-dependent probe amplification; G6PD, glucose-6-phosphate dehydrogenase; CNS, central nervous system; Hb, hemoglobin; FGR, fetal growth restriction; NGS, next-generation sequencing.

## Data Availability

No new data were created or analyzed in this study. Data sharing is not applicable to this article.

## Abbreviations

AC	Abdominal circumference
BWS	Beckwith–Wiedemann syndrome
CMA	Chromosomal microarray analysis
CMV	Cytomegalovirus
CVS	Chorionic villus sampling
ECCI	European Congenital Cytomegalovirus Initiative
EPO	Erythropoietin
ERT	Enzyme replacement therapy
FGR	Fetal growth restriction
Hb	Hemoglobin
HDFN	Hemolytic disease of the fetus and newborn
HIV	Human immunodeficiency virus
HLH	Hemophagocytic lymphohistiocytosis
ISUOG	International Society of Ultrasound in Obstetrics and Gynecology
IUERT	In utero enzyme replacement therapy
IUT	Intrauterine transfusion
LCH	Langerhans cell histiocytosis
LSD(s)	Lysosomal storage disease(s)
MCA-PSV	Middle cerebral artery peak systolic velocity
NGS	Next-generation sequencing
NIHF	Non-immune hydrops fetalis
NIPT	Noninvasive prenatal testing
NK cells	Natural killer cells
PCR	Polymerase chain reaction
PMD	Placental mesenchymal dysplasia
SCT	Sacrococcygeal teratoma
SIFD	Sideroblastic anemia with B-cell immunodeficiency, periodic fevers, and developmental delay
SMFM	Society for Maternal–Fetal Medicine
TAM	Transient abnormal myelopoiesis
TAPS	Twin anemia–polycythemia sequence
TORCH	Toxoplasmosis, Other, Rubella, Cytomegalovirus, Herpes
TRAb	Thyrotropin receptor antibodies
TRAP	Twin reversed arterial perfusion
TSI	Thyroid-stimulating immunoglobulins
VUS	Variants of Uncertain Significance
WES	Whole-exome sequencing
WGS	Whole-genome sequencing
